# Monitoring and evaluating an implementation strategy aimed at improving interprofessional collaboration in community-based fall prevention: a mixed-methods study

**DOI:** 10.1186/s43058-025-00814-w

**Published:** 2025-11-21

**Authors:** Meike C. van Scherpenseel, Lidia J. van Veenendaal, Di-Janne J. A. Barten, Cindy Veenhof, Marielle H. Emmelot-Vonk, Saskia J. te Velde

**Affiliations:** 1 Research Group Innovation of Human Movement Care, Research Center for Healthy and Sustainable Living, HU University of Applied Sciences Utrecht, Utrecht, 3501AA The Netherlands; 2 Research Group Proactive Care for Older People Living at Home, Research Center for Healthy and Sustainable Living, HU University of Applied Sciences Utrecht, Utrecht, 3501AA The Netherlands; 3 Bachelor of Nursing, Institute for Nursing Studies, HU University of Applied Sciences Utrecht, Utrecht, 3501AA The Netherlands; 4 Department of Rehabilitation, Physiotherapy Science and Sport, University Medical Center Utrecht, Utrecht, University, Utrecht, 3508GA The Netherlands; 5 Center for Physical Therapy Research and Innovation in Primary Care, Julius Health Care Centers, De Meern, 3454PV The Netherlands; 6 Department of Geriatrics, University Medical Center Utrecht, 3508GA Utrecht, The Netherlands

**Keywords:** Monitoring, Evaluation, Implementation strategy, Interprofessional Collaboration, Community, Fall prevention

## Abstract

**Background:**

Interprofessional collaboration (IPC) among health and social care providers is crucial to effectively implement community-based fall prevention. Several factors hinder successful and sustainable IPC, highlighting the need to both design and evaluate context-specific implementation strategies. However, there remains a fundamental gap in the detailed description and evaluation of such strategies. Therefore, this study aims to (1) monitor the implementation process over time and (2) evaluate the impact of a multifaceted implementation strategy aimed at improving interprofessional collaboration among health and social care professionals in community-based fall prevention.

**Methods:**

This study was conducted in two districts and one municipality in the Netherlands. We conducted a longitudinal mixed-methods study with a convergent design, emphasizing qualitative methodology. Over 24 months, qualitative (focus groups and regular meetings) and quantitative (questionnaires) data were collected semi-annually from three working groups of health and social care professionals (HSCPs). Qualitative and quantitative data were initially analyzed separately, followed by an integrated analysis for comprehensive insights on themes influencing the implementation process and the impact of the strategy on IPC and implementation outcomes.

**Results:**

In total, 32 HSCPs originating from three communities participated in this study. Monitoring and evaluation of the multifaceted implementation strategy revealed four overarching themes: (1) “Network building”, including aspects and activities that contribute to network building; (2) “Team dynamics”, referring to interactions within the working groups; (3) “Coordination”, addressing the coordination of implementation and establishment of protocols and work flows; and (4) “Implementation dynamics” highlighting aspects that influence the implementation process and outcomes.

**Conclusions:**

This study identified four key themes influencing the implementation process and impact of a multifaceted implementation strategy aimed at improving IPC among HSCPs in community-based fall prevention: network building, team dynamics, coordination and implementation dynamics. Monitoring and evaluation are crucial for identifying the specific activities needed to effectively implement interventions in real-world settings. Given the complexity of implementation processes and ongoing contextual changes, continuous adjustments are necessary. An iterative monitoring and evaluation approach, as used in this study, enables these adaptations and maximizes real-world impact.

**Supplementary Information:**

The online version contains supplementary material available at 10.1186/s43058-025-00814-w.

Contributions to the literature

- This study is the first to comprehensively monitor and evaluate a multifaceted implementation strategy aimed at improving interprofessional collaboration (IPC) in community-based fall prevention, offering practical insights and deepen the understanding of mechanisms underlying successful implementation.
- This study emphasizes the importance of continuous, iterative monitoring and evaluation to adapt strategies to dynamic contexts, ensuring that implementation efforts remain relevant and effective in real-world settings.
- Four key themes - network building, team dynamics, coordination, and implementation dynamics - were identified that influence the implementation process of strategies to strengthen IPC in community-based fall prevention and have impact on both IPC and implementation outcomes.

## Background

More than a third of the community-dwelling older adults over 65 years of age experience one or more falls each year [1]. Falls can lead to significant health impact such as morbidity, disability, hospitalization and mortality, leading to substantial healthcare costs [1, 2]. The incidence of falls and fall-related injuries are expected to increase due to the aging society, making the prevention and management of falls a crucial public health priority worldwide [3–5].

Falls often result from a combination of modifiable risk factors, such as gait and balance issues, poor vision, the use of fall risk increasing medications and home hazards [1, 6]. Extensive research supports a multifactorial approach to assess and manage these risk factors, reducing falls among older adults and improve the general health of the population [1, 7]. These holistic approaches involves referring to various health and social care services for tailored evidence-based interventions, such as exercise programs, medication reviews and addressing home fall-hazards [1, 7, 8]. This highlights the need for effective interprofessional collaboration (IPC) among different health and social care providers [6, 9]. Despite such evidence, fall rates have remained largely unchanged for years, with poor implementation of fall prevention knowledge into routine practices being an important contributing factor [10–12]. In response to this issue, there is growing interest in applying implementation science to fall prevention, focusing on how to effectively implement fall prevention knowledge and adopt evidence-based interventions in everyday practice [13, 14].

Evidence from implementation science consistently emphasizes the complexity of knowledge translation, highlighting the role of contextual factors influencing this process - often called contextual (implementation) determinants or barriers and facilitators [15]. One of the most reported contextual barriers to successful implementation of fall prevention practices is poor IPC [6, 9, 16–18]. IPC refers to the collaboration of multiple professionals from different professional backgrounds to provide services by working with patients, their families, and the community, to provide the highest quality of care [19]. Effective IPC is essential to meet the health needs of communities [19–21]. However, several factors hinder successful IPC in community-based fall prevention. These include inexplicitly defined roles and responsibilities among professionals, inadequate communication and coordination, and limited interpersonal relationships within multidisciplinary teams [6, 9, 22, 23]. On the other hand, role clarification, interdependence, team functioning, collective ownership of goals, and collaborative leadership facilitate IPC [6, 9, 19, 22]. Professionals also need to acquire necessary competencies for successful collaboration [20].

After identifying barriers and facilitators, the next step in implementation processes is to design implementation strategies that address them [24]. In current literature, implementation strategies are often poorly described, missing operational definitions or guidelines for their use in real-world settings [25, 26]. There is a growing call for implementation studies to provide more detailed descriptions of the strategies employed, including specifics on actions, roles and timelines [25–27]. This can be done by monitoring or tracking strategies and their implementation process over time, which facilitates a better evaluation of their effectiveness and deepens understanding of underlying working mechanisms [27].

Despite the importance of IPC, there remains a persistent gap in the detailed description and evaluation of strategies aimed at improving IPC, particularly in fall prevention. To address this gap, this study aims to (1) monitor the implementation process over time and (2) evaluate the impact of a multifaceted implementation strategy aimed at improving interprofessional collaboration among health and social care professionals in community-based fall prevention on both IPC and implementation outcomes.

## Methods

### Design and setting

This study is part of the larger, multi-disciplinary implementation study FRIEND (Fall pRevention ImplEmeNtation stuDy, 2020-2024), which aimed to implement fall prevention programs in the community. The FRIEND-study took place in two districts of different large cities and one small municipality in the area of Utrecht, the Netherlands. The study was approved by the Ethical Committee Research Healthcare Domain of the HU University of Applied Sciences, Utrecht, the Netherlands (113-000-2020) and all participants consented to participate.

In this sub-study of FRIEND, we conducted a 24-month longitudinal mixed-methods study with a convergent design with a focus on qualitative methodology. We used this design to examine the implementation process and the strategy’s impact on IPC and implementation outcomes. Qualitative focus groups with diverse community-related health and social care professionals (HSCPs) provided in-depth process insights, while quantitative questionnaires at four time points (T0-T3) offered complementary trends. Integrating both methods enabled a deeper understanding than either method alone [28]. Reporting followed the Standards for Reporting Implementation Studies (StaRI) checklist (Appendix 1) [29].

### Participants

At the start of the FRIEND-study, various HSCPs working with fall prevention (such as general practitioners, physiotherapists, occupational therapists, and pharmacists) were included. Inclusion criteria were: (1) health or social care professional involved in community-based fall prevention efforts, and (2) working in either one of the districts or the municipality. There were no exclusion criteria. Multidisciplinary working groups were created in the two districts (working groups 1 and 2) and municipality (working group 3). The current study was performed within these three working groups. We specifically focused on provider-provider collaboration, as major barriers to effective IPC in multifactorial fall prevention persist in this area [6, 9, 30, 31].

### Study procedure

In the planning of the FRIEND-study, we drew upon the enhanced Replicating Effective Programs (REP) framework. It supports outlining a package of implementation strategies to be executed across four phases of implementation: ‘Pre-condition’, ‘Pre-implementation’, ‘Implementation’ and ‘Maintenance and evolution’ [26, 32].

The Pre-condition phase was performed prior to the current study: contextual determinants to the implementation of multifactorial community-based fall prevention interventions were identified in focus groups in each working group. To complement findings, a literature review was performed and combined results, using the constructs and domains from the Consolidated Framework for Implementation Research (CFIR) [33], were described in a scoping review and published elsewhere [16].

#### Pre-implementation phase

We mapped implementation strategies based on prior insights from the focus groups. This enabled us to design strategies that were optimally aligned with their context. Strategy mapping was conducted in co-creation with the HSCPs in the three working groups, by using the original CFIR-Expert Recommendations for Implementing Change (ERIC) Implementation Strategy Matching Tool [34, 35]. The process was performed in several steps. First, per working group, we entered the identified barriers into the tool, which then generated a prioritized list of recommended implementation strategies. Second, in online co-creation sessions with the HSCPs in each working group, we presented the top ten strategies recommended by the tool and the HSCPs selected their personal top three. Among all three working groups, there was an overlap in the strategies identified as most important, all of which were related to IPC. These included: ‘*building a coalition*’, ‘*conduct educational meetings*’, ‘*organize clinician implementation team meetings*’, ‘*identify and prepare champions’* and ‘*promote network weaving*’.

Eventually, consensus in all working groups was reached on one multifaceted strategy, defined as: ‘improving interprofessional collaboration among health and social care professionals in community-based fall prevention’.

#### Implementation phase

The Implementation phase involved the execution of the multifaceted implementation strategy. This phase was set up in three steps. First, each working group received an online Interprofessional Collaboration-training. The training was performed by two researchers from the FRIEND Research Group to support HSCPs in exploring their local interprofessional network [20, 21], and defining concrete implementation activities related to the implementation strategy which were included in an action plan. Second, all working groups started with the implementation of the strategy within their community by executing the implementation activities in the action plan. Third, the researchers (MS, JB, SV) developed an iterative monitoring and evaluation plan for data collection to be carried out every six months. The aim of *monitoring* was to monitor and track the implementation activities outlined in the action plan (i.e. how, by whom and when they were executed) to identify what influenced the implementation process; *evaluation* focused on assessing the impact of these activities on IPC and implementation outcomes. Iteration and intermediate insights enabled us to - in collaboration with the working groups - continuously refine the implementation strategy and activities in the action plans to the context, when needed.

### Data collection

Monitoring the implementation process and evaluation of the impact of the implementation strategy took place during the Implementation phase, semi-annually, from November 2021 (baseline, T0) until November 2023 (T3). Since the working group in the municipality started later with the execution of implementation activities, we only collected their data from baseline until T2. Study outcomes and the mode of their collection are presented in Table 1.

**Table 1:** Outcomes and procedure for data collection

**Outcome**	**Qualitative (focus groups and regular meetings)**	**Quantitative (questionnaires)**
		Background characteristics
Monitoring the implementation process	Logbook and meeting notes (Fidelity of the implementation strategy)	
Evaluation of the impact of the implementation strategy on IPC	Logbook, meeting notes, and barriers and facilitators	• CICS29: competencies regarding interprofessional collaboration • TCI: team climate • SNA: network dynamics
Evaluation of the impact of the implementation strategy on implementation outcomes		• AIM: acceptability of the implementation strategy • IAM: appropriateness of the implementation strategy • FIM: feasibility of the implementation strategy

*Abbreviations*: *AIM* Acceptability of intervention measure, *IAM* Intervention appropriateness measure, *FIM* Feasibility of intervention measure, *CICS29* Chiba interprofessional competency scale, *TCI* Team climate inventory, *SNA* Social network analysis

#### Qualitative data collection

Qualitative data collection consisted of semi-annual focus groups. The focus groups were designed following the Dynamic Learning Agenda (DLA) methodology [36]. The DLA is an iterative method to facilitate reflection and track progress of concrete short-term activities, which were updated in the action plan [36]. Each focus group was organized and led by one researcher, another researcher observed and took meeting notes. In addition, the working groups held regular meetings every 6–12 weeks. Regular meetings were led by the working group’s chair. The researchers were not actively involved in the discussions.

##### Monitoring the implementation process

To monitor the implementation process, we collected data by taking meeting notes about the current status of implementation activities (i.e. are they executed (Fidelity), if not why, who is in charge) during the focus groups and regular meetings [37]. Additionally, a logbook was kept to record notable findings that occurred during the meeting, including who made decisions or took a leading role and how this was done, group atmosphere, and members’ reactions to each other and their ideas. At the end of each focus group and regular meeting, the action plan was updated by deleting finished activities and adding new ones. The action plan was maintained by both the researchers - to track progress of activities - and the HSCPs - to remind which actions to execute.

##### Evaluation of the impact of the implementation strategy

To evaluate the impact of the strategy on IPC, we longitudinally collected barriers and facilitators. These determinants were examined as process indicators of IPC, with changes over time to provide explanatory insight into how the implementation influenced IPC. During the focus groups, participants documented barriers and facilitators on post its, which were subsequently managed in Excel for analysis.

#### Quantitative data collection

A questionnaire was distributed semi-annually among the HSCPs in the working groups. The general part collected background characteristics including age, gender, educational level, profession, work setting, working experience, working mono- and/or multi-disciplinary. Five existing questionnaires were added to monitor the process and evaluate the impact of the implementation strategy.

##### Evaluation of the impact of the implementation strategy

To evaluate the impact of the implementation strategy on IPC we operationalized IPC in terms of three key elements: network development, team work and interprofessional competencies [20, 38]. Also, we evaluated the impact of the strategy on implementation outcomes: Acceptability, Appropriateness and Feasibility of the implementation strategy [37].

- Social Network Analysis
  A Social Network Analysis (SNA) survey was distributed to gain insight in network development over time per working group [39, 40]. There are no standardized questions for an SNA, so we drew from examples in the literature [ 39–41]. The final SNA included questions on with whom participants collaborated regarding fall prevention (number of contacts). With this data, we could indicate if reciprocity occurred between participants who filled out the questionnaire. After feedback from participants on the questions, we changed the questionnaire slightly for T2 and T3, allowing participants to choose if they had two-sided collaboration with another professional, or only made referrals (one-sided collaboration) and if they received referrals in return from that professional. This provided more detailed data on reciprocity.
- Team Climate Inventory
  The development of team climate within the working groups over time was measured with the self-reported Dutch Team Climate Inventory (TCI) [42, 43]. The Dutch TCI is validated for multidisciplinary patient care-teams. The TCI is a 38-item questionnaire, answered on a 5-Point Likert scale (1 = completely disagree; 5 = completely agree), and has four subscales: Vision, Participative safety, Task orientation and Support for innovation.
- Chiba Interprofessional Competency Scale
  A Dutch version of the self-reported Chiba Interprofessional Competency Scale (CICS29) was used to longitudinally assess whether the HSCPs possessed competencies related to IPC [45]. The CICS29 consists of 29 items, categorized into six subscales: Attitudes and beliefs as a professional (ABP), Team management skills (TMS), Actions for accomplishing team goals (ATG), Providing care that respects patients (PCRP), Attitudes and behaviors that improve team cohesion (ABTC) and Fulfilling one’s role as a professional (FRP). Each item was scored on a five-point Likert scale (1 = completely disagree; 5 = completely agree).
- Implementation outcomes: Acceptability, Appropriateness, Feasibility
  These outcomes were measured using the Acceptability of Intervention Measure (AIM), Feasibility of Intervention Measure (FIM) and Appropriateness of Intervention Measure (IAM) [46]. Each instrument has four items and scoring options on a 5-point Likert scale (1 = completely disagree; 5 = completely agree). This English questionnaire was translated to Dutch using the forward-backward translation method [47].

### Data analysis

**Fig. 1:**
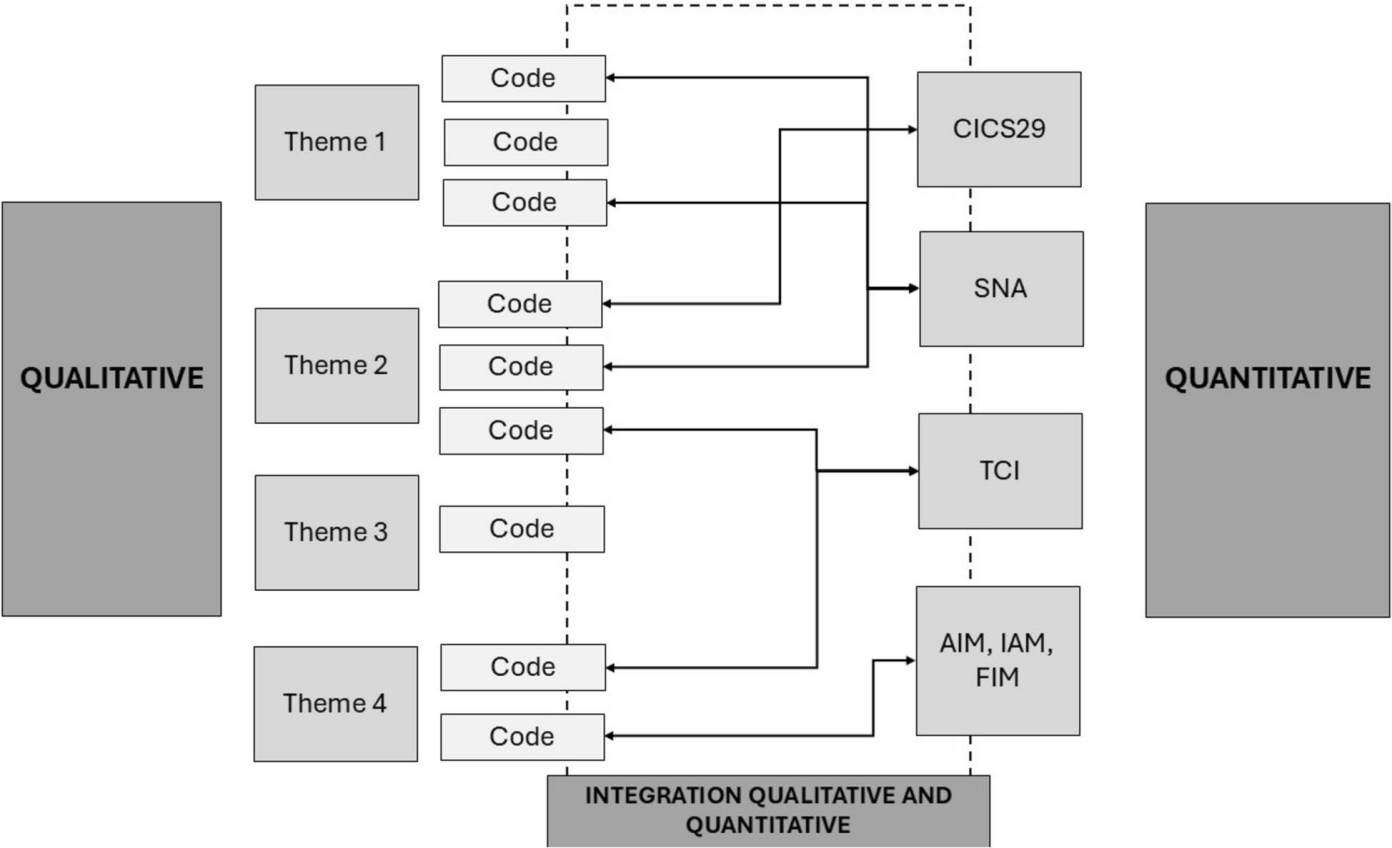
Integration of qualitative and quantitative data analysis. Abbreviations: AIM = Acceptability of Intervention Measure; IAM = Intervention Appropriateness Measure; FIM = Feasibility of Intervention Measure; CICS29 = Chiba Interprofessional Competency Scale; TCI = Team Climate Inventory; SNA = Social Network Analysis

The qualitative and quantitative data were initially analyzed separately, followed by an integration (Fig. 1).

#### Qualitative data analysis

Qualitative data were analyzed using a hybrid thematic analysis [48, 49]. We started by inductively open coding the meeting notes, logbooks and Excel-documents (n = 32 documents) using Atlas.ti (version 24)®. We used data-driven coding and only information regarding the implementation strategy under study was coded [50]. This was performed independently for one working group by two researchers (MS and LV). Afterwards, these researchers met in a consensus meeting to create a coding scheme and to search for connections between open codes to form categories (axial coding), which were organized into a preliminary set of overarching themes.

Furthermore, as deductive element, the coding scheme was supplemented in various ways. First, by components of a theoretical model for IPC (e.g. role clarity, collaborative leadership, communication) [22]. Second, the implementation outcome Fidelity was added to the coding scheme. Fidelity can be analyzed by measuring three aspects: adherence, dose, and participant responsiveness [51]. Third, outcomes which were quantitatively measured (AIM, IAM, FIM, SNA, CIC29 and TCI) were either connected to existing codes or newly added to the coding scheme.

One researcher (MS) used the coding scheme to code the remaining documents. Inductive open codes which did not fit the coding scheme either expanded a predetermined code or were added as new. Afterwards, both researchers met again to define definite overarching themes.

#### Quantitative data analysis

Quantitative data were analyzed using IBM SPSS Statistics, 29.0.0.0®. Background characteristics were analyzed using descriptive statistics (counts, median, interquartile range, percentages). The SNA was analyzed using NodeXL, an open-source basic social network analysis tool in Microsoft Excel [52]. Network development was determined by computing the number of contacts and reciprocity, and was visualized in social network diagrams [40]. To measure competencies regarding IPC among the HSCPs over time, the total and subscale mean scores of the CICS29 were calculated. To assess the evolution of team climate over time, measured by the TCI, the mean score per subscale was computed. The TCI means were interpreted as follows: Strongly disagree in the point range of 1.00–1.79.00.79; Disagree 1.80–2.59.80.59; Neutral 2.60–3.39.60.39; Agree 3.40–4.19.40.19; and Strongly agree 4.20–5.0.20.0 [53]. To calculate Acceptability, Appropriateness and Feasibility scores for the multifaceted implementation strategy, the mean scores of the AIM, IAM and FIM were summed up (scoring range 4–20points for each measure) [54].

To analyze the changes in scores of these questionnaires over time, the scores of the three working groups were combined. This approach was chosen due to the small number of HSCPs per time point and because all working groups were working on the same multifaceted strategy. Combining data provides a more robust overview of the findings over time.

#### Integration of qualitative and quantitative results

After separate analysis, results of the qualitative findings were compared and differences, similarities, contradictions are relationships were identified with quantitative findings [28]. The utilization of multiple data sources helped to validate findings and enhanced credibility [55]. We specifically looked where qualitative findings could be linked to quantitative findings and vice versa, and whether they supported each other or not.

Integrated results are presented in a narrative discussion, by organizing the results within the same theme derived from the qualitative analysis. Regarding the CICS29 and TCI, only the subscales which seemed to be related to IPC after integration of the results were described in the Results. Eventually, we presented our findings to involved HSCPs to enhance the level of confirmability and to ensure objectivity [55].

## Results

### Participants

In total, 32 HSCPs participated in this study divided over three working groups. The composition of the working groups varied over time; some HSCPs entered the study at the start of the FRIEND-study but dropped-out and others were included at a later point in time. Reasons for dropping out were: other work-related priorities, sick or maternity leave and/or working only limited with the target population and therefore not feeling the urge to participate. Characteristics of the participants are shown at the point they entered the study (Table 2). Almost all participants were female. Diverse professions were present, of which most participants were physiotherapist (*n *= 7), nurse practitioner-somatic health care (*n* = 4) or podiatrist (*n* = 4). The majority of the participants worked in a primary health center or practice (*n* = 23). While most participants worked multidisciplinary, this varied across the working groups.

**Table 2:** Baseline characteristics

Characteristic	Working group 1 (*N* = 10)	Working group 2 (*N* = 12)	Working group 3 (*N* = 10)
Age (*n*)			
- < 25 years-35 years - 36–45 years - 46–55 years - 56–65 years	*n* = 4 *n* = 2 *n* = 1 *n* = 3	*n* = 3 *n* = 3 *n* = 3 *n* = 3	*n *= 3 *n* = 2 *n* = 2 *n* = 3
Gender, female (%)	100%	91.7%	100%
Educational level (*n*)			
- Secondary vocational education - Higher professional education, Bachelor’s degree - Higher professional education, Master’s degree - Academic education	- *n* = 5 *n* = 2 *n* = 3	- *n* = 7 *n* = 2 *n* = 3	*n *= 2 *n* = 3 *n* = 2 *n* = 2
Profession (*n*)			
- Clinical nurse specialist - Community nurse - Community sports coach - Dietician - General practitioner - Nurse practitioner - mental health care - Nurse practitioner - somatic health care - Occupational therapist - Pharmacist - Physiotherapist - Podiatrist - Policy officer, public health service	*n* = 2 *n* = 1 - - *n* = 1 - *n* = 1 *n* = 1 - *n* = 3 *n* = 1 -	- - *n* = 1 *n* = 2 - *n* = 1 *n* = 3 - *n* = 1 *n* = 1 *n* = 2 *n* = 1	- *n* = 2 *n* = 1 *n* = 1 - - - *n* = 1 *n* = 1 *n* = 3 *n* = 1 -
Work setting (*n*)			
- Community/municipality - Primary health care center/practice* - Hospital - Nursing home	- *n* = 9 *n* = 1 -	*n* = 2 *n* = 10 - -	*n* = 1 *n* = 7 - *n* = 2
Working experience in years, median (IQR)	3.5 (2.5–13.3.5.3)	14.0 (9.3–20.5.3.5)	21.0 (2.5–26.3.5.3)
Working mono-disciplinary, yes (%)	50%	80%	66.7%
Working multi-disciplinary, yes (%)	90%	60%	83.3%

*Abbreviations*: *IQR* Interquartile range

^*^For example: physiotherapist practice, general practitioner practice, dietetic practice, pharmacy, nursing care organization

### Integrated findings per theme

Integrated analysis revealed four overarching themes influencing the implementation process and the impact of the strategy on IPC and implementation outcomes. Theme 1: “Network building” includes aspects and activities that contribute to network building. Theme 2: “Team dynamics” refers to dynamics within the working groups. Theme 3: “Coordination” focuses on the coordination and establishment of protocols and work flows. Theme 4: “Implementation dynamics” relates to aspects influencing the ongoing implementation process and outcomes. These themes operate at multiple levels, both internal (team, organization) and external (policy, researcher engagement). Continuous contextual adaptations were crucial to align the strategy with local circumstances, address current barriers, and maximize impact overall. The implementation strategy is described in detail following Proctor’s guidelines (Appendix 2) [25].

#### Theme 1: Network building

An important aspect to improve IPC was network building. One key implementation activity derived from qualitative data was creating a social care map. This helped HSCPs to get to know each other and each other’s role, which facilitated purposeful collaboration and referrals. Ideally updated regularly and available online, this map provides information on professionals working in the neighborhood, their roles in fall prevention and how to contact them. Typically, a professional with a generalist background, who interacts with a wide range of HSCPs, led the creation and updating of the map.

*“One of the things to make sure they know each other better is to create a social care map. […] Given that there are many personnel changes in the district, it [should be] updated* *regularly and indicate who to contact in the district. This will lead to stable collaboration.” -* Working group 2.

Additionally, organizing in-person meetings regularly, lasting at least one hour and held every 6 to 12 weeks at accessible and familiar locations (e.g. general practitioner practice) was identified in the qualitative data to improve IPC. These meetings helped to exchange knowledge regarding each other’s role.

*“The goal of the networking meeting is mainly for disciplines to know each other better and to strengthen collaboration.”* - Working group 2.

**Fig. 2:**
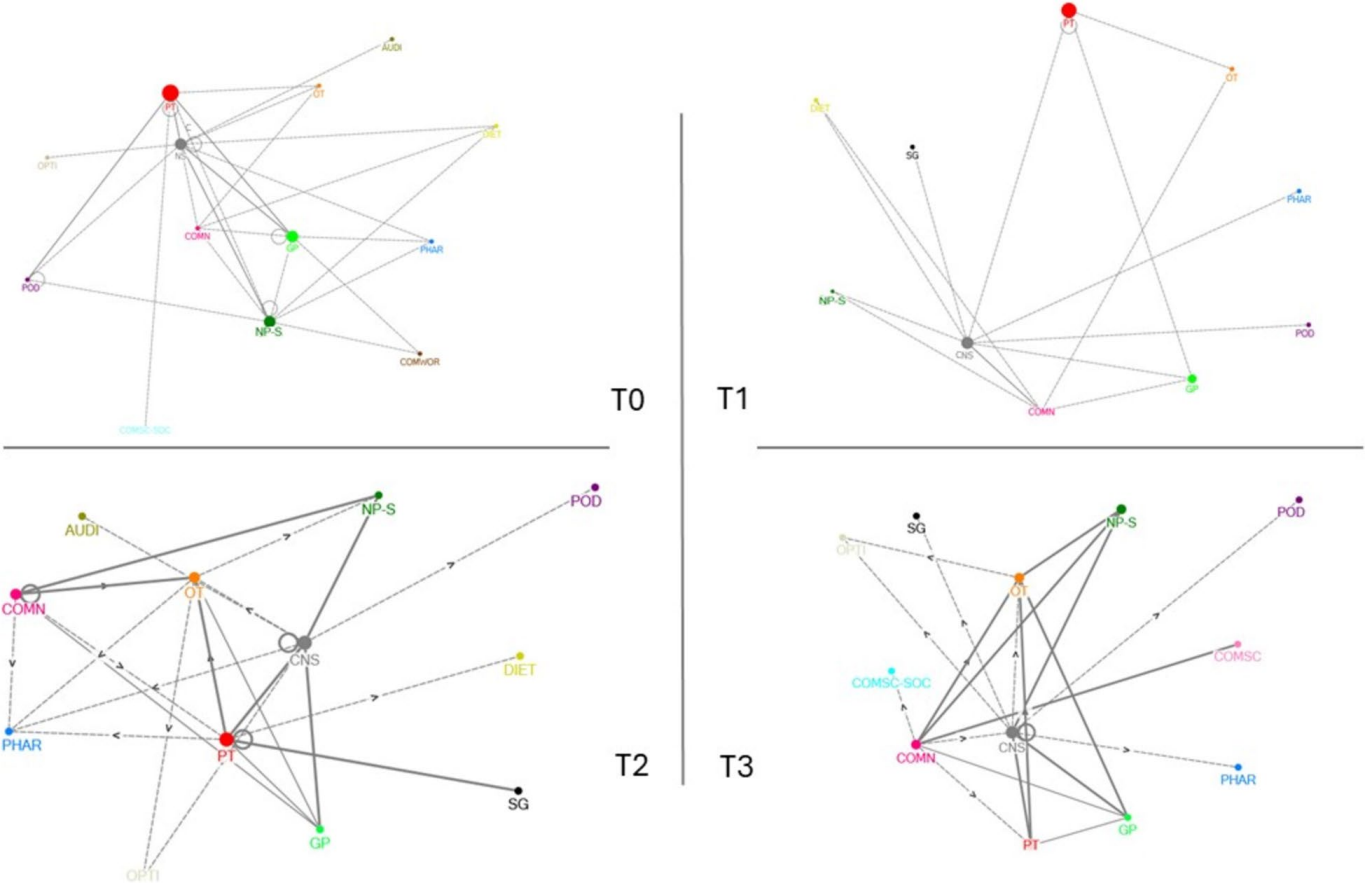
Social network diagrams T0-T3, Working Group 1. Abbreviations: •PT = physical therapist; •GP = general practitioner; •OT = occupational therapist; •DIET = dietician; •PHAR = pharmacist; •POD = podiatrist; •COMN = community nurse; •NP-S = nurse practitioner-somatic health care; •CNS = clinical nurse specialist; •COMSC-SOC = community sports coach (social); •COMSC = sommunity sports coach; •COMNWOR = community worker; •OPTI = optician; •AUDI = audiologist; •SG = specialist geriatrics

Results from the SNA showed that such activities helped to expand local networks. The social network diagrams show that both the number of contacts among HSCPs and the reciprocity improved over time (Fig. 2). It is notable that for some disciplines the number of contacts differed across timepoints. Also, the disciplines to whom professionals referred or with whom they collaborated, differed among the working groups. For example, the clinical nurse specialist was mentioned as collaborating discipline often in one working group, in comparison to the others. Qualitative findings revealed that certain individuals were important for network stability due to their active involvement while others played a more passive role, but this was regardless of their profession.

Furthermore, qualitative findings indicated that engaging various professionals, specifically linking pins, improved IPC. Linking pins are professionals who collaborate with multiple other professionals. HSCPs frequently mentioned the need to connect with linking pins, typically initiated by a generalist HSCP. Social network diagrams revealed that physiotherapists, nurse practitioners, community nurses, general practitioners and occupational therapists often had several interprofessional contacts. They were considered to play a vital role in referring older adults to the right discipline, although this varied across the working groups.

#### Theme 2: Team dynamics

**Fig. 3:**
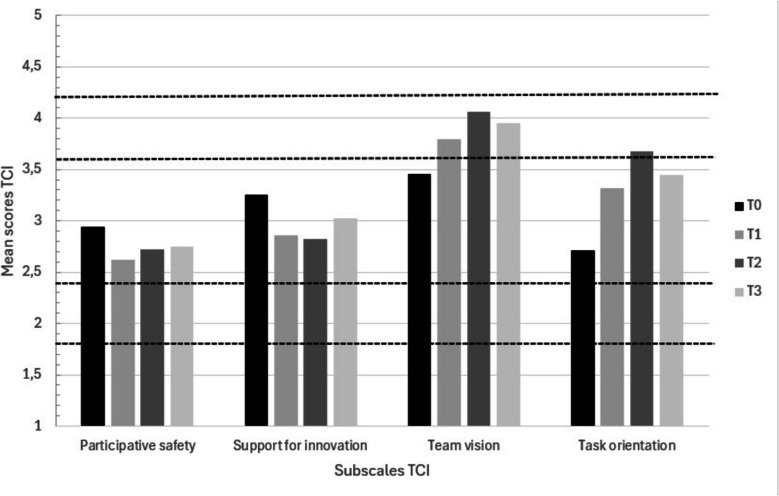
Mean scores of the subscales of the Team Climate Inventory across the working groups over time. The dotted line indicates the categorization to interpret the results: 1.00–1.79.00.79 = strongly disagree; 1.80–2.39.80.39 = disagree; 2.40–3.59.40.59 = neutral; 3.60–4.19.60.19 = agree; 4.20–5.0.20.0 = strongly agree

Various elements related to the team dynamics were deemed important in improving IPC. First, qualitative findings revealed that setting a common goal at the start of the working groups was essential in improving IPC as this increased the feeling of working together, having a good atmosphere within the team, and feeling interdependent of each other. Consistently, the TCI results indicated that the Team Vision subscale scored highest among all working groups and showed the largest improvement over time (Fig. 3).

**Fig. 4:**
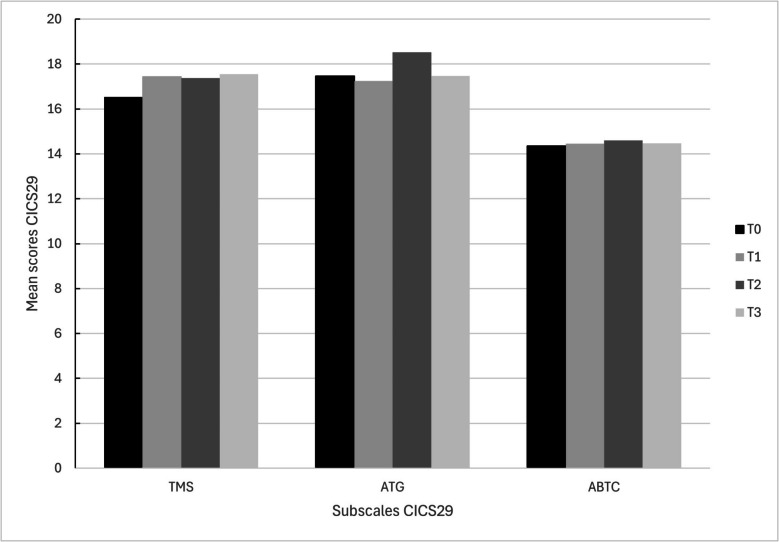
Mean scores of the subscales of the Chiba Interprofessional Competency Scale (CICS29) across working groups over time. Abbreviations: TMS = Team Skills Management; ATG = Actions for accomplishing team goals; ABTC = attitudes and behaviors that improve team cohesion

Also, qualitative data revealed that having a sense of clarity of objectives to which all participants committed improved IPC. They executed activities to accomplish goals, which were concrete and summarized at the end of each meeting so everyone knew what to do. They felt competent in executing them according to the findings of the subscale ‘Actions for accomplishing team goals’ of the CICS29 (Fig. 4).

*“The meeting ends with a division of concrete action points when it comes to creating the social care map, and checking how existing networks react on bringing up the topic ‘fall prevention’. The division of actions […] allows everyone to proceed effectively.”* - Working group 1.

Qualitative findings revealed that a positive working environment, including everyone being enthusiastic and feeling motivated, improved IPC as it made it more fun to collaborate with each other. However, TCI’s subscale ‘Participative safety’ was generally scored within the neutral range and remained similar over time (Fig. 3), which may be attributed to some participants who were heavily involved in decision-making, dismissing others and others ideas, as identified in the qualitative data.

Furthermore, having well-developed skills and attitudes regarding IPC, including trusting each other, being committed and being flexible, were mentioned as supportive for IPC within the team. The working groups seemed to self-learn how to effectively work together within the team over time; this was reflected in the subscale ‘Team management skills’ of the CICS29 which developed slightly over time (Fig. 4).

Also, qualitative data revealed that working groups were taking time to discuss with each other on new ideas. Participants took the time to finalize implementation activities to a high standard, ensuring their effectiveness, as shown by activities in the action plans remaining ‘active’ for a long time. The feeling of working collaboratively on high-quality outputs was reflected in the relatively high scores on the TCI’ subscale ‘Task orientation’ (Fig. 3). Overall these scores increased over time, and participants expressed satisfaction with the cooperative execution of the project.

#### Theme 3: Coordination

Qualitative findings highlighted coordination as key to improving IPC. Especially appointing a local coordinator who provides leadership and ensures activities are executed as planned. A ‘linking pin’ was suggested for this role due to their central position in the local network. This individual should work independently and oversee and connect similar activities and (existing) community networks, fostering efficient and collaborative working.

However, frequent changes in coordinators within each working group and inconsistent follow-up resulted in team members lacking motivation to execute activities as no one put pressure on it.

*“There needs to be a coordinator, someone driving the project. It should be someone who can keep an eye on all processes in the neighborhood, so we don’t get stuck on our own ‘piece’.” -* Working group 3.

Another activity to improve IPC which was derived from the qualitative data was creating a referral pathway. In this pathway, agreements among professionals were made, which led to an unequivocal working method and more referrals.

*“It is important to set out an unequivocal line[…] which can easily be implemented by others. This way, everyone is working the same way, according a uniform policy. The referral pathway makes the referral structure clearer.” -* Working group 2.

#### Theme 4: Implementation dynamics

Qualitative data showed that successful implementation was dependent on contextual aspects, such as financial restraints and changes in external policy, and continuous adaptation of the strategy seemed essential to fit local, dynamic circumstances. It was suggested as crucial to regularly gain insight into contextual determinants to align activities accordingly.

Another aspect that impacted the implementation was the active involvement of the researchers of the FRIEND-study. They organized - as planned - semi-annual focus groups, which helped the team to meet frequently, discuss the process, and identify new activities or address activities that had not yet been executed properly.

*“During the implementation, the meetings of FRIEND were really facilitating. This was* *another moment to get together; these have always given a boost [to the implementation] as well.”*—Working group 3.

**Fig. 5:**
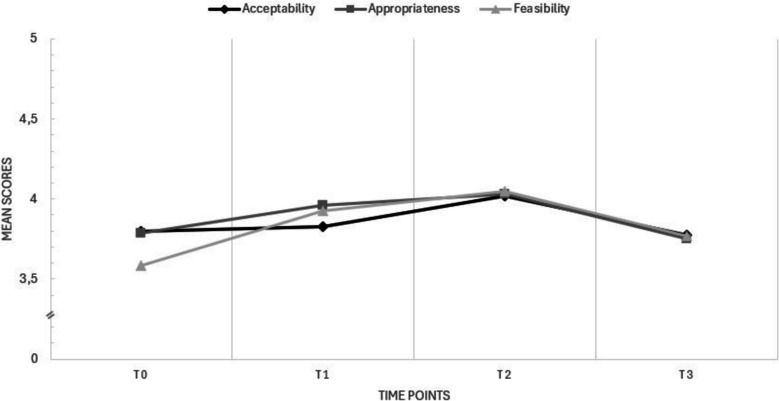
Mean scores Acceptability (AIM), Appropriateness (IAM) and Feasibility (FIM) over time across the working groups

Furthermore, qualitative data revealed that the implementation strategy was considered appropriate and acceptable as it fitted well with experienced barriers regarding IPC at the start of the study. In line with these findings, the quantitatively measured Acceptability and Appropriateness increased over time across the working groups until T2 (Fig. 5).

*“Working collaboratively is important but not going well, if you look at how many disciplines could be involved.”*—Working group 3.

Also, the strategy was considered feasible to some extent at the start, but a few factors were mentioned to influence the changes in IPC. Especially limited time and money, high workload, lack of support from management and frequent personnel changes seemed to affect IPC negatively. Although some of these factors remained, quantitative scores of feasibility increased slightly over time (Fig. 5).

Quantitative analysis revealed a decrease in mean scores for all three implementation outcomes after T2. In the final focus group, qualitative data indicated that some professionals were satisfied with the current IPC and their focus shifted to other barriers, particularly uncertainty regarding financial opportunities due to significant changes in national policy and funding for fall prevention.

Regarding ‘adherence’ of fidelity of the implementation strategy, it was noticed that most implementation activities were executed but not always according to plan, mostly due to time constraints and the deviations of involved team members. It helped professionals to distribute small tasks among each other.

*“Execution of tasks occurs very often in ‘my own time’ or in quickly in between consults with patients, so the pace gets stuck. Dividing tasks among several people is a solution; we need to do it with the entire group.”* - Working group 1.

Furthermore, regarding ‘dose’ of fidelity, qualitative analyses revealed that not all participants were present at each meeting, which made it difficult to make solid agreements that in the end applied to everyone.

Regarding ‘participant response’ of fidelity*,* participants were fully engaged in the development of the implementation strategy. This was performed during the Pre-implementation phase; based on their selection, the final implementation strategy was drafted. Implementation activities were developed by the participants in the working groups themselves.

## Discussion

This study aimed to monitor the process and evaluate the impact of a multifaceted implementation strategy aimed at improving IPC among HSCPs in community-based fall prevention on both IPC and implementation outcomes. To our knowledge, no previous research has focused on this topic. Our findings highlight that four overarching themes influence the implementation process and the impact of an implementation strategy to improve IPC: network building, team dynamics, coordination and implementation dynamics.

A key element for improving IPC is the development and maintenance of networks. In multifactorial fall prevention, such networks are essential for coordinating care, enhancing patient outcomes and improving efficiency [6, 56]. Optimally, they extend beyond health providers to include older adults, family, (in)formal caregivers, and the wider community [22] - as their involvement is crucial for tailoring fall prevention interventions [57].

While much existing literature on networks is descriptive [58], this study adds detailed insights into activities that improved network building in multifactorial fall prevention, based on close strategy monitoring. Our findings suggest that networks are often fragile and dependent on individuals rather than professions. This aligns with previous research showing that while multidisciplinary teams usually emphasize professional background, networks actually represent a set of individuals with varying attributes, demographics and attitudes [59, 60]. Promoting sustainable networks therefore requires identifying and engaging key individuals rather than focusing solely on specific professional roles.

In addition to networks, another form of interprofessional practice is teamwork [38], as demonstrated within the working groups of this study [61–63]. While elements of effective teamwork have been described [61–63], optimal approaches to strengthen team functioning remain largely unclear [63, 64]. Our findings provide detailed suggestions, such as ensuring all members to have a sense of clarity by summarizing agreed actions and allocating time for discussion, which may also inform other complex community-based interventions. Both the IPC-training and the DLA-methodology contributed to creating concrete implementation activities and enhancing overall team performance. Also, team climate, and in particular participative safety, was found to contribute to higher team functioning, since safety influences shared decision-making [65]. Participants reported that a positive working environment was crucial to facilitate effective collaboration within their teams.

Furthermore, coordination emerged as a crucial factor for enabling effective professional interaction. This has been highlighted in previous research as well, suggesting that such coordinators hold a key role in establishing and maintaining teams [22, 66, 67]. These key players can be identified as ‘linking pins’, i.e. those who most optimally span the network [59]. In fall prevention, this role is typically assigned to professionals in addition to their regular duties, which makes networks vulnerable when they rely too heavily on a single central figure who have a major central role in holding a network together [66]. As observed in all working groups, the departure of such key players can compromise network stability. To mitigate this risk, appointing an external coordinator already active in implementing fall prevention or designating at least two coordinators may strengthen sustainability [67].

Moreover, implementation efforts are inherently influenced by dynamic contextual changes, adding complexity [68, 69]. In our study, quantitative data showed that the strategy for improving IPC became less acceptable and appropriate over time. This decline may be attributed to contextual changes such as internal team dynamics (e.g. team composition changes, inconsistent team members attendance) and external factors (e.g. changes in fall prevention policy). These findings underscore the importance to iteratively monitor, evaluate and adapt implementation strategies allowing for real-time adjustments that account for ‘real-world’ conditions [68, 70]. This approach helps to align efforts with emerging issues and promoting sustainable implementation over time.

This study has several strengths. First, we conducted a practice-oriented implementation study, supporting HSCPs in real-time improvements to their daily practice, directly bridging the gap between research and practice [71, 72]. Second, the use of a longitudinal mixed-methods approach enabled iterative monitoring and evaluation, providing detailed insights into and understanding of the implementation process, including what was done, by whom and what may have impacted this process. We aligned with key priorities in implementation science that call for more detailed descriptions of strategies [27]. Looking beyond implementation, there is a need for more longitudinal studies that specifically investigate sustainability and scaling up implementation, while focusing on implementation processes. Such research remains rare but represents an essential area for future research that contributes to more effective interventions that has sustainable impact on population health [68].

There are several limitations to this study. First, capturing the complex relationships between contextual factors and their impact on the implementation process is generally challenging in implementation research, including this study. Despite efforts to understand and adapt to the dynamic context, pinpointing what specifically influenced the implementation process remained difficult. This highlights a persistent gap in understanding how context precisely shapes implementation strategies, including how they work and why, for example by following adaptations and creating causal pathways for identifying underlying mechanisms. This remains an area for future research, resulting in both scientific knowledge and practical guidance [70, 73]. Second, we combined questionnaire data across all working groups, except for the SNA, after individual results seemed similar - probably due to all working groups following a similar implementation process. Combining the data increased robustness and power of our findings, particularly given the small sample sizes. Third, we did not conduct statistical testing to assess the significance of observed changes due to variability in respondents and small sample sizes. Instead, we present the findings as descriptive trends, which should be considered when interpreting the results. Fourth, the active involvement of researchers of the study appeared to facilitate improvements in IPC, as we supported in-person meetings and the execution of implementation activities. It is known that implementation researchers often find themselves as partners in implementation projects, enhancing both implementation practices and evaluation [71]. However, in real-world settings, such involvement is not feasible, suggesting the need for other roles such as Implementation Support Practitioners: a relatively new profile for professionals supporting organizations, professionals and leaders in applying implementation science in daily practices [74, 75].

## Conclusions

Monitoring and evaluating implementation strategies are essential for identifying which activities should be applied to effectively implement interventions, who is responsible them, and their impact in real-world settings. Our findings highlight four key themes that influence the implementation process and the impact of an implementation strategy aimed at improving IPC among HSCPs in community-based fall prevention: network building, team dynamics, coordination and implementation dynamics. However, implementation processes are often complex, which is enlarged by ongoing contextual changes. To consistently align with the evolving context, continuous adjustments are necessary during implementation. An iterative monitoring and evaluation approach, as used in this study, fits best to enable adaptations, maximizing real-world impact.

## Supplementary Information

Supplementary Material 1.

Supplementary Material 2.

## Data Availability

The quantitative datasets supporting the conclusions of this article are available in the DataverseNL repository (10.34894/PS75Q2) and are available from the corresponding author on reasonable request. The qualitative dataset supporting the conclusions of this article are not publicly available due to privacy reasons, but are available from the corresponding author on reasonable request.

## Abbreviations

IPC: Interprofessional collaboration
FRIEND: Fall pRevention ImplEmeNtation stuDy
HSCPs: Health and social care professionals
STARI: Standards for Reporting Implementation Studies
REP: Replicating Effective Programs
CFIR: Consolidated Framework for Implementation Research
CFIR-ERIC: CFIR-Expert Recommendations for Implementing Change
DLA: Dynamic Learning Agenda
AIM: Acceptability Intervention Measure
IAM: Appropriateness of Intervention Measure
FIM: Feasibility of Intervention Measure
SNA: Social Network Analysis
TCI: Team Climate Inventory
CICS29: Chiba Interprofessional Competency Scale, 29 items
