# Supplementary material for: Monitoring and evaluating an implementation strategy aimed at improving interprofessional collaboration in community-based fall prevention: a mixed-methods study
Source: Implement Sci Commun. 2025 Nov 21;6:128. doi: 10.1186/s43058-025-00814-w (PMC12639693; doi:10.1186/s43058-025-00814-w)
Supplement: Supplementary file 1 — Supplementary Material 1. [file 43058_2025_814_MOESM1_ESM.docx]

**Appendix 1. Standards for Reporting Implementation Studies (StaRI) checklist**

| **Checklist item** | | **Reported on page #** | **Implementation Strategy** | **Reported on page #** | **Intervention** |
| --- | --- | --- | --- | --- | --- |
|  | |  | “Implementation strategy” refers to how the intervention was implemented |  | “Intervention” refers to the healthcare or public health intervention that is being implemented. |
| **Title and abstract** | | | | | |
| Title | **1** | 1 | Identification as an implementation study, and description of the methodology in the title and/or keywords | | |
| Abstract | **2** | 2 | Identification as an implementation study, including a description of the implementation strategy to be tested, the evidence-based intervention being implemented, and defining the key implementation and health outcomes. | | |
| **Introduction** | | | | | |
| Introduction | **3** | 5 | Description of the problem, challenge or deficiency in healthcare or public health that the intervention being implemented aims to address. | | |
| Rationale | **4** | 5-6 | The scientific background and rationale for the implementation strategy (including any underpinning theory/framework/model, how it is expected to achieve its effects and any pilot work). |  | The scientific background and rationale for the intervention being implemented (including evidence about its effectiveness and how it is expected to achieve its effects). |
| Aims and objectives | **5** | 6 | The aims of the study, differentiating between implementation objectives and any intervention objectives. | | |
| **Methods: description** | | | | | |
| Design | **6** | 6 | The design and key features of the evaluation, (cross referencing to any appropriate methodology reporting standards) and any changes to study protocol, with reasons | | |
| Context | **7** | 6-7 | The context in which the intervention was implemented. (Consider social, economic, policy, healthcare, organisational barriers and facilitators that might influence implementation elsewhere). | | |
| Targeted ‘sites’ | **8** | 6-7 | The characteristics of the targeted ‘site(s)’ (e.g locations/personnel/resources etc.) for implementation and any eligibility criteria. |  | The population targeted by the intervention and any eligibility criteria. |
| Description | **9** | 7-8 | A description of the implementation strategy |  | A description of the intervention |
| Sub-groups | **10** | 7 | Any sub-groups recruited for additional research tasks, and/or nested studies are described | | |
| **Methods: evaluation** | | | | | |
| Outcomes | **11** | 8-14 | Defined pre-specified primary and other outcome(s) of the implementation strategy, and how they were assessed. Document any pre-determined targets |  | Defined pre-specified primary and other outcome(s) of the intervention (if assessed), and how they were assessed. Document any pre-determined targets |
| Process evaluation | **12** | 8-9 | Process evaluation objectives and outcomes related to the mechanism by which the strategy is expected to work | | |
| Economic evaluation | **13** | n/a | Methods for resource use, costs, economic outcomes and analysis for the implementation strategy |  | Methods for resource use, costs, economic outcomes and analysis for the intervention |
| Sample size | **14** | 14 | Rationale for sample sizes (including sample size calculations, budgetary constraints, practical considerations, data saturation, as appropriate) | | |
| Analysis | **15** | 11-14 | Methods of analysis (with reasons for that choice) | | |
| Sub-group analyses | **16** | n/a | Any a priori sub-group analyses (e.g. between different sites in a multicentre study, different clinical or demographic populations), and sub-groups recruited to specific nested research tasks | | |

| **Results** | | | | | |
| --- | --- | --- | --- | --- | --- |
| Characteristics | **17** | 14-15 | Proportion recruited and characteristics of the recipient population for the implementation strategy |  | Proportion recruited and characteristics (if appropriate) of the recipient population for the intervention |
| Outcomes | **18** | 15-24 | Primary and other outcome(s) of the implementation strategy |  | Primary and other outcome(s) of the Intervention (if assessed) |
| Process outcomes | **19** | 15-24 | Process data related to the implementation strategy mapped to the mechanism by which the strategy is expected to work | | |
| Economic evaluation | **20** | n/a | Resource use, costs, economic outcomes and analysis for the implementation strategy |  | Resource use, costs, economic outcomes and analysis for the intervention |
| Sub-group analyses | **21** | n/a | Representativeness and outcomes of subgroups including those recruited to specific research tasks | | |
| Fidelity/ adaptation | **22** | 23-24 | Fidelity to implementation strategy as planned and adaptation to suit context and preferences |  | Fidelity to delivering the core components of intervention (where measured) |
| Contextual changes | **23** | 15-24 | Contextual changes (if any) which may have affected outcomes | | |
| Harms | **24** | 15-24 | All important harms or unintended effects in each group | | |
| **Discussion** | | | | | |
| Structured discussion | **25** | 24-27 | Summary of findings, strengths and limitations, comparisons with other studies, conclusions and implications | | |
| Implications | **26** | 24-27 | Discussion of policy, practice and/or research implications of the implementation strategy (specifically including scalability) |  | Discussion of policy, practice and/or research implications of the intervention (specifically including sustainability) |
| **General** | | | | | |
| Statements | **27** | 28-30 | Include statement(s) on regulatory approvals (including, as appropriate, ethical approval, confidential use of routine data, governance approval), trial/study registration (availability of protocol), funding and conflicts of interest | | |
