# Supplementary material for: Monitoring and evaluating an implementation strategy aimed at improving interprofessional collaboration in community-based fall prevention: a mixed-methods study
Source: Implement Sci Commun. 2025 Nov 21;6:128. doi: 10.1186/s43058-025-00814-w (PMC12639693; doi:10.1186/s43058-025-00814-w)
Supplement: Supplementary file 2 — Supplementary Material 2. [file 43058_2025_814_MOESM2_ESM.docx]

**Appendix 2. Description of the implementation strategy following Proctor’s guidelines.**

| **Name it** | | | | | | | |
| --- | --- | --- | --- | --- | --- | --- | --- |
| Improving interprofessional collaboration among health and social care professionals in community-based fall prevention. | | | | | | | |
| **Define it** | | | | | | | |
| Establish, coordinate and maintain interactive, interprofessional collaborative relationships among various health and social care professionals in the context of implementing community-based fall prevention. | | | | | | | |
| **Specify it** | | | | | | | |
| **Actor** | **Individual element of implementation strategy*** | **Action** | **Action target** | **Temporality** | **Dose** | **Outcomes affected** | **Justification** |
| HSCPs working in community-based fall prevention included in the FRIEND-study in the working groups | 1. Network building | 1. Creating a social care map 2. Organizing in-person meetings 3. Engage various professionals | Build collective ownership and relationships; understanding each other’s role | At start of Implementation phase, continuously | 1. One time and then ongoing as needed 2. Minimum of 1-hour meeting, every 6-12 weeks; variability in dose per working group 3. One time and then ongoing as needed | Larger network among HSCPs (SNA) | Building a coalition, conduct educational meetings, organize clinician implementation team meetings, promote network weaving, identify and prepare champions (CFIR) |
|  | 1. Team dynamics | 1. Setting a common goal 2. Having a sense of clarity 3. Positive working environment 4. Having well-developed skills and attitudes IPC 5. Taking time to discuss | Build interdependence, commitment, competences | At start of Implementation phase, continuously | One time, and then ongoing as needed | Improved team climate (TIC) and competencies IPC (CICS29) |  |
|  | 1. Coordination | 1. Appointing a local coordinator 2. Creating a referral pathway | 1. Provide collaborative leadership 2. Developing an unequivocal working method | 1. At start of Pre-implementation phase, continuously 2. At start of Implementation phase, continuously | One time and then ongoing as needed | Appropriate leadership, making agreements |  |
|  | 1. Implementation dynamics | 1. Gain insight in contextual determinants      1. Active involvement of researchers 2. Fidelity | 1. Align implementation activities 2. Support and facilitate implementation process 3. Consistent execution of implementation activities according to plan by engaged HSCPs | 1. Semi-annually and iteratively throughout Implementation phase 2. Continuously throughout Implementation phase 3. Continuously throughout Implementation phase | 1. Half hour with each working group 2. Regularly, variability in dose by working group 3. Ongoing, check during meetings | Contextual determinants; Acceptability (AIM), Appropriateness (IAM), Feasibility (FIM), Fidelity |  |

Abbreviations: HSCPs = health and social care professionals; CFIR = Consolidated Framework for Implementation Research; IPC = interprofessional collaboration * identified themes in the current study.
